# Contextual Emotions in Organizations: A Latent Profile Analysis of Their Co-Occurrence and Their Effects on Employee Well-Being

**DOI:** 10.3390/ejihpe15070122

**Published:** 2025-07-02

**Authors:** Laura Petitta, Lixin Jiang, Valerio Ghezzi

**Affiliations:** 1 Department of Psychology, Sapienza University of Rome, Via dei Marsi, 78, 00185 Rome, Italy; valerio.ghezzi@uniroma1.it; 2 School of Psychology, University of Auckland, 23 Symonds Street, Auckland 1010, New Zealand; l.jiang@auckland.ac.nz

**Keywords:** contextual emotions at work, emotion-related patterns of workplace behavior, latent profile analysis, employee well-being, job satisfaction

## Abstract

Workplace contextual emotions are structured ways of emotionally thinking about specific cues in the context that employees share within their organization. These dynamics reflect how employees emotionally interpret and respond to organizational environments. Contextual emotions may shape working relationships into different types of toxic emotional dynamics (e.g., claiming, controlling, distrusting, provoking) or, conversely, positive emotional dynamics (i.e., exchanging), thus setting the emotional tone that affects employees’ actions and their level of comfort/discomfort. The present study uses latent profile analysis (LPA) to identify subpopulations of employees who may experience differing levels of both positive and negative emotional dynamics (i.e., different configurations of emotional patterns of workplace behavior). Moreover, it examines whether the emergent profiles predict work-related (i.e., job satisfaction, burnout) and health-related outcomes (i.e., sleep disturbances, physical and mental health). Using data from 801 Italian employees, we identified four latent profiles: “functional dynamics” (low toxic emotions and high exchange), “dialectical dynamics” (co-existence of medium toxic emotions and medium exchange), “mild dysfunctional dynamics” (moderately high toxic emotions and low exchange), and “highly dysfunctional dynamics” (extremely high toxic emotions and extremely low exchange). Moreover, employees in the dialectical, mild dysfunctional, and highly dysfunctional groups reported progressively higher levels of poor health outcomes and progressively lower levels of satisfaction, whereas the functional group was at low risk of stress and was the most satisfied group. The theoretical and practical implications of the LPA-classified emotional patterns of workplace behavior are discussed in light of the relevance of identifying vulnerable subpopulations of employees diversely exposed to toxic configurations of emotional/relational ambience.

## 1. Introduction

The literature, based on various epistemological frameworks and theoretical perspectives, suggests that emotional ambience in organizations sets the tone of social interactions among members and related patterns of working behavior, thus underpinning employees’ experience of comfort, or discomfort, and satisfaction in regard to the quality of their working life (Ashkanasy & Dorris, 2017). Poor interpersonal relations or a lack of practices and policies related to respect for workers are psychosocial hazards within an organization and significantly contribute to workplace stress (Stoewen, 2016), whereas positive social relationships among employees allow the work to get done. The overarching goal of this research is to study the emotional foundations of action in organizations. To reach this goal, on one side, we propose a conceptual approach that goes beyond the more common intra-individual dispositional approach to contextual emotional factors. On the other side, we utilize an interactionist approach to the explanation of workplace behaviors, which encompasses the interplay between emotional, cognitive, and contextual factors.

The present study focuses on contextual emotions at work, understood as structured ways of emotionally thinking about specific cues in the context that employees share within their organization and that may shape working relationships into different types of toxic emotional dynamics (e.g., claiming, provoking, controlling, distrusting) or, conversely, positive emotional dynamics (i.e., exchanging). These dynamics reflect how employees emotionally interpret and respond to organizational environments. As such, they set the social–emotional tone that affects employees’ actions, as well as their sense of comfort/discomfort. Specifically, our goal is three-fold. Firstly, we examine the emotional foundations of action in organizations by using an interactionist approach encompassing the interplay between emotion, cognition, and context, and go beyond the intra-individual dispositional approach to contextual emotional factors. Secondly, we use a latent profile approach to emotion-related interpersonal factors in order to identify subpopulations of employees who may differ in the way that they emotionally symbolize their workplace and, therefore, how they perform daily nurturing and constructive (or, conversely, maladaptive) behaviors that contribute to structure a comfortable relational ambience, underpinning healthy work environments. We used this methodological approach because latent profile analysis is by definition an exploratory technique that attempts to uncover groups of subpopulations based on how different appraisals (levels) of a construct (e.g., contextual emotions) tend to co-occur in regard to employees and, thus, can be used to identify different configurations of the construct’s profiles (Oberski, 2016). As such, rather than focusing on predetermined combinations of variables of interest, we examine how these combinations of variables tend to emerge and characterize different groups of people among organizational members. In doing so, we lay the basis for the identification of differently vulnerable groups of employees in terms of their exposure to toxic emotional environments. Thirdly, we assess the role of latent profiles of emotion-related interpersonal factors gained through socialization among organizational members in predicting employee well-being and job satisfaction.

First, we propose an interactionist paradigm to workplace emotional/relational dynamics, grounded in the conjoint interplay between cognition, emotion, and context in regard to explaining behavior. As noted, *contextual emotions at work* are emotional symbolization processes through which the human mind assigns emotional importance (emotion) to each represented element (cognition) of an experience within a specific workplace and social environment (context). These contextual emotions are unwantedly shared among employees and reinforced by the network of socialization practices (Carli & Paniccia, 2003). Thus, recurrent exposure to the environment (e.g., social interactions) helps form structured ways of emotionally thinking about contextual cues. This process shapes emotion-related schemes of thinking and acting that are widespread among the community (contextual emotions or, rather, workplace emotional dynamics) and define the emotional atmosphere in the workplace (Ashkanasy & Dorris, 2017; Carli & Paniccia, 2003; Petitta et al., 2018). In doing so, our conceptualization of contextual emotions at work differs from approaches that consider contextual emotions solely as contextual information in situations to help people make sense of their feelings (e.g., Arellano et al., 2015; Guo et al., 2018). The novel conceptualization proposed in the current study is grounded in the Theory of Analysis of Demand (Carli & Paniccia, 2003) and complements existing emotion theories (e.g., affective events theory; see Ashkanasy & Dorris, 2017 for a review). Contextual emotions are different from well-established emotion-related concepts and phenomena. Specifically, the literature on emotions in organizations (e.g., Barsade & Gibson, 2007) suggests that affect represents an umbrella term encompassing a broad range of emotion-related phenomena, including short-term (e.g., feeling state, mood) or dispositional long-lasting (e.g., trait affectivity) feelings that employees experience. While the literature on emotions (e.g., Barsade & Gibson, 2007; Grandey et al., 2013) includes different constructs, such as discrete emotions, moods, trait affect, emotional intelligence, emotional regulation, emotional labor, and collective affect (i.e., the degree to which individual levels of affective characteristics combine to form group level emotions), what they all have in common is an intra-individual approach to the experience of emotion-related phenomena. That is, whether short term or long lasting, affect is framed from the perspective of the inner experience of the individual, even when elicited by an external cause. Conversely, workplace contextual emotions are mental representations of one’s own context that are immersed in the emotions shared among the people interacting at work, which compel employees to structure a relational pattern of interaction that is fueled by emotions that are mutually exchanged. As such, contextual emotions represent an *inter-individual* approach to affect in the workplace, as opposed to the renowned *intra-individual* approaches. The inter-individual approach to contextual emotions also complements and is different from similar context-focused approaches to emotions at work. For example, affective events theory (AET; Weiss & Cropanzano, 1996) posits that employees’ affective experiences are reactions to work effects and are also activated by situational causes. In comparing contextual emotions with traditional theories, such as AET, a main point of departure is that AET calls attention to the role of environmental features as proximal causes of an affective reaction. Conversely, contextual emotions direct attention away from emotions as an intra-individual experience and a mere reaction to a trigger, and shift the attention towards an interactionist perspective of the mental representations of one’s own context that are socially co-created by the mutually satisfying emotional needs of people interacting at work.

Workplace contextual emotions also differ from other macro-level emotion-related constructs, such as emotional culture (Barsade & O’Neill, 2014), collective emotional aperture (Sanchez-Burks & Huy, 2009), and collective emotion regulation (Gross, 2015). Specifically, emotional culture refers to widespread emotions at the collective level among employees through contagion and captures the deep underlying assumptions about the meaning of emotions (e.g., compassionate love). Consistently, it is measured by focusing on “reporting the expressed (not felt) emotions of other employees” (Barsade & O’Neill, 2014, p. 567). While emotional culture consists of the expression of emotions at the collective level, contextual emotions refer to collective relational dynamics that are fueled by different types of emotional experiences. In a similar vein, “emotional aperture entails a person’s ability to recognize the dynamic emotional composition of a collective” and refers to a one-time perception of employees (Sanchez-Burks & Huy, 2009, p. 27). As such, it departs from contextual emotions that entail the perception of specific patterns of relational dynamics that are grounded in different emotional experiences. Finally, the combined relational and emotional features of contextual emotions make them different from the construct of collective emotion regulation, which is mainly concerned with the collective rules that shape the expression of emotions and their trajectory over time (Gross, 2015).

Second, to date, existing research has mainly adopted a variable-centered strategy to understand the impact of positive or toxic emotional experiences on variables of interest. However, such an approach does not account for the fact that individuals may simultaneously experience differing levels of both positive (i.e., exchange) and toxic (e.g., provoke, control, distrust) emotional dynamics in the workplace. We resolve this issue by first examining the emergence of different configurations of emotional patterns of workplace behavior, using latent profile analysis (LPA; Morin et al., 2018). Specifically, using LPA, we first aim to identify distinct profiles of employees, based on their levels of positive and toxic emotional patterns of workplace behavior. Given that the difference between positive and toxic emotions depends on both their quantity (i.e., intensity) and their quality (David et al., 2005), it is necessary to use LPA to reveal quantitatively (i.e., levels) and qualitatively (i.e., combinations) distinct configurations of emotional patterns of workplace behavior (i.e., positive and toxic workplace emotional dynamics).

Third, the current literature on predictors of employee well-being tends to separately examine the roles of (a) relational and emotional processes and (b) individual, interpersonal, and organizational/contextual factors (e.g., Hati & Pradhan, 2021; Sonnentag et al., 2023). To complement existing theoretical perspectives, our proposal on contextual emotions at work that conjointly examines the interplay of individual (e.g., cognition, emotion) and contextual factors (Carli & Paniccia, 2003), enables us to provide a holistic approach to understand whether emotional profiles of positive and toxic emotional dynamics at work may relate to work-related and health outcomes.

Below, we begin our review of the literature by addressing the concept of contextual emotions at work. Next, we provide a theoretical background on latent profile analysis applied to contextual emotions, and the relationship between emergent contextual emotion profiles and employee well-being outcomes (i.e., emotional exhaustion, cynicism, sleep disturbances, physical health, mental health), as well as job satisfaction.

## 2. Theoretical Background: Contextual Emotions at Work and the Latent Profile Approach

Indeed, many models of emotions hypothesize that emotions and cognition are intertwined (Ashkanasy & Dorris, 2017). However, they diversely place the emphasis on the primacy of conscious evaluation (i.e., cognition) in regard to the immediate affective response (i.e., emotion) (e.g., Lazarus, 1982), or on the neural basis of their interplay, as in the case of research (e.g., LeDoux, 2002) suggesting that the activation of the brain areas involved in producing an emotional reaction (i.e., limbic system) precedes that associated with cognition (i.e., neocortex). Moreover, flourishing theories on emotions and contextual factors posit that emotions are situated phenomena that prepare the organism to manage a given situation (e.g., Pugh et al., 2022). It is noteworthy that they diversely model the context as (a) an environmental setting that affects how emotions are experienced depending on how people conceptualize the situation (e.g., Wilson-Mendenhall et al., 2011), or (b) as the organizational environment constellated by events to which employees affectively react (e.g., affective events theory; Weiss & Cropanzano, 1996), or (c) as national cultural beliefs and norms that vary regarding the features ascribed to an emotion and, thus, influence emotional behaviors, perceptions, and experiences (Kitayama et al., 2006; Markus & Kitayama, 1991).

The novel conceptualization of contextual emotions proposed in the current study is rooted in the Theory of Analysis of Demand (TAD; Carli & Paniccia, 2003), engaging an interactionist (individual–context) and holistic model of mind functioning that thoroughly addresses the conjoint interplay of cognition, emotion, and context in explaining organizational behavior and related emotion-driven relational dynamics. The key conceptual points are at least three-fold. First, people’s experience of their interaction with their context is abstracted (i.e., cognitive process) and associated with an emotional experience (i.e., emotional process). Consequently, the human mind attaches emotional value to every contact and experience within their context, and the real outside context is replaced with an emotional mental representation of it. Second, the theory posits that an individual’s behavior is the result of how they emotionally interpret their relationship within their context. As such, it represents an interactionist (individual–context) approach, which integrates both the cognitive and emotional functioning of the mind in order to explain organizational behavior. Third, repeated experiences within a context shared with other people contribute to structuring the interpersonal dynamics that reflect how people emotionally interpret and respond to organizational environments, thus shaping the emotional ambience they are exposed to on an ongoing basis. As noted, while renowned collective-level emotion theories tend to frame affect from the perspective of the inner experience of the individual, the experience of emotion in collective settings (e.g., organizations) proposed by the TAD refers to shared mental representations of the context that compel employees to structure a relational pattern of interaction that is fueled by emotions that are mutually exchanged. The key differences are the type of emotions at stake (e.g., discrete emotions vs. socially intertwined emotional exchanges) and the mutual reinforcement of the relational pattern. For example, a converging view on positive relationships at work suggests that positive interactions are actions that confer a sense of value and worth to others and depend on respect, openness, and connectedness (Lee et al., 2019). Conversely, the positive emotional patterns of workplace behavior (e.g., exchanging dynamics) proposed by the TAD are relational dynamics fueled by a person’s emotional openness to others and their active exploration of reality (one party), counteracted by the sharing of information (other party), that reiterates mutual exchanges, encourages curiosity in regard to each other, and instills mental calmness when interacting with each other. In organizational settings soaked with exchanging dynamics, employees do not take each other for granted, are not scared about getting to know new people, nor do they experience hostility towards anything new, and they mutually search for dialog as a source of reciprocal knowledge.

The basic tenets that underpin the proposed conceptualization are: (1) the bi-logic mind functioning framework, (2) the individual-in-context approach, (3) collusion, (4) the relationship with the reality/context (known vs. unknown), and (5) positive and toxic contextual emotions.

First, the *bi-logic* functioning of the mind (Matte Blanco, 1975) refers to a model of the mind that includes two levels of information processing (i.e., one cognitive and one emotional) and maintains that the human mind processes one’s interaction with their context through two different yet intertwined levels: the *unconscious* and the *conscious*. Thus, behavior arises from the interaction between two modes of mental operation: conscious thought organizes the cues from the context based on heterogeneous and dividing principles, while the unconscious level processes the context according to generalization and symmetry principles or, rather, emotional logic. Both levels play a role in shaping an individual’s interactions with their environment.

Second, the individual-in-context approach proposes that an individual is an entity that must be considered within a given context, while the context is an abstraction of reality and, therefore, a mental representation. The individual–context relationship becomes an emotionally symbolized mode of mental functioning, where an emotional mental representation replaces the real outside context. Hence, emotional symbolization describes the process whereby an individual connects their encounters in the external environment with an unavoidable emotional response.

Third, people socialize their emotional environment through interaction. *Collusions,* from the Latin “*cum-ludere*”, or, rather, playing together in a relationship, are dynamics that people are unaware of that enable one to meet the complementary needs of other people within organizations (Carli & Paniccia, 1981). For example, people who need affiliation may spontaneously and involuntarily go along with people who need to exert power over others, and the reverse. Therefore, collusions guide individuals to organize and emotionally construct their reality, share similar or commentary emotional symbols, and foster an interpersonal and belonging structure based on socially shared emotional experiences.

Fourth, encountering any individual in the workplace unveils an “unknown side”, even when we have feelings of familiarity, due to shared similarities. When one party views the other as an unknown entity, it prompts active engagement to uncover their potential contributions to the relationship, fostering information exchange and emotional bond exploration. Conversely, when people take reality “for granted”, they fail to notice information or take deceitful shortcuts to save energy and reduce anything novel to something familiar that can be ignored.

Fifth, since birth, the human mind tends to categorize into different compartments the information that it gets from its experience in certain situations. According to the TAD, the human mind addresses the *unknown* side of reality (i.e., potentially novel information) by progressively developing more evolved forms to emotionally organize it (i.e., put the information into different emotion-related categories or rather, “emotional boxes”). The TAD proposes two main ways to emotionally symbolize the “unknown” side of the organizational context (i.e., contextual emotions): (1) a toxic, dysfunctional way and (2) a positive, functional way. The difference between the two types of contextual emotions depends on how the individual tends to relate to the “unknown” side of reality. Specifically, toxic ways of emotionally symbolizing reality may “close” peoples’ minds towards anything new or unknown (e.g., uncertain situations), thereby compelling all individuals sharing the same context to emotionally avoid novelties associated with change or diversity. Conversely, the positive way of emotionally symbolizing reality may “open” peoples’ minds towards the unknown, which requires a positive approach and exploration, and cannot be taken for granted. While there exists only one positive way to relate, the theory warns against multiple different negative ways to emotionally relate within organizations, each conducive to different patterns of toxic emotional dynamics within communities.

It is noteworthy that employee socialization practices involuntarily contribute to the collusion process, fostering toxic (or wholesome), yet persistent, emotional ways to relate that set the tone of the emotional atmosphere of daily working life. In other words, collusive processes in organizations are the glue that maintains the stability of contextual emotions. Importantly, discrete emotions (e.g., fear, anger; Ekman, 1972) and emotional drives (e.g., psychological insatiability; Dweck, 2000; Vallerand, 2012) are the propellants that feed social interactions and the structured pattern of working relationships.

**Figure 1:**
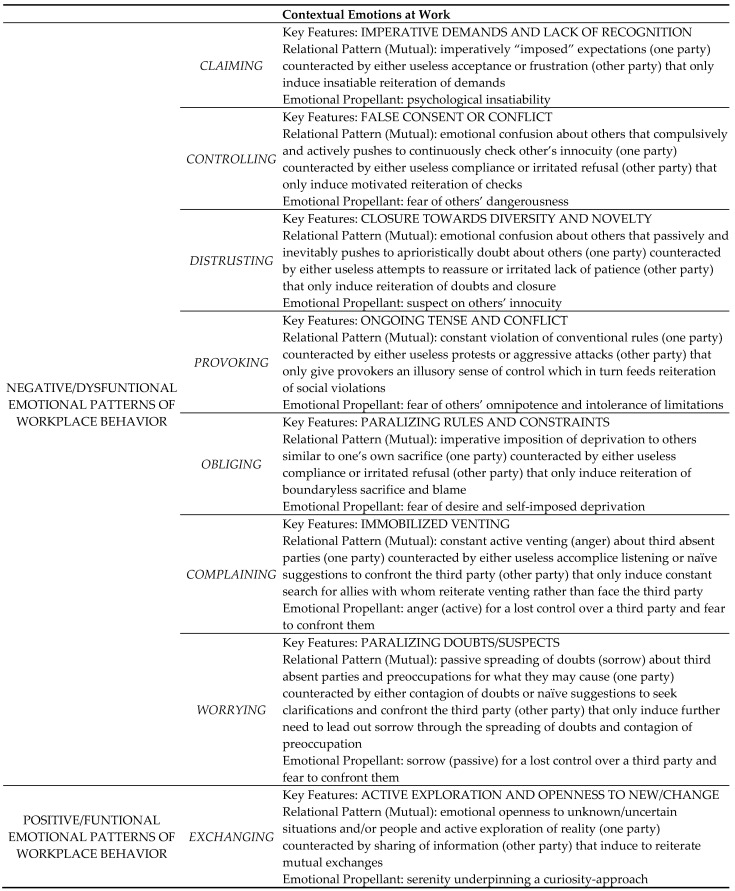
Description of the positive and negative contextual emotions at work.

Building on the above theoretical background, we propose the following positive contextual emotion at work, namely *exchanging dynamics*, and seven toxic contextual emotions at work, namely *claiming dynamics*, *controlling dynamics*, *provoking dynamics*, *distrusting dynamics*, *obliging dynamics*, *complaining dynamics*, and *worrying dynamics*. Below we provide details for each type of contextual emotion: (a) the specific types of emotions that feed working relationships, and (b) the resulting emotional patterns of workplace behavior that structure relational dynamics. Figure 1 provides a simplified model of the emotional dynamics for each contextual emotion and describes the relevant contextual emotional patterns.

*Claiming* is the contextual emotion structured around asymmetrical relationships among parties that is rooted in the emotional experience of imperative demands and a frustrating lack of recognition. In working environments with relational schemes based on a “claim”, the following pattern of behaviors are structured. Some people imperatively demand “something” (e.g., respect, understanding, priority, obedience) by referring to the role that they have (e.g., boss, user who pays taxes, seriously ill person), based on their presumed importance or superiority over others, thereby emotionally experiencing their role as a way to obtain others’ dependence. Others, in turn, eagerly indulge demanding individuals, despite a constant lack of recognition, thus feeding working relationships based on dysfunctional emotions of insatiability (demanders) and frustration (pleasers).

*Controlling* is characterized by asymmetrical relationships among parties that are rooted in the fear of others’ dangerousness and active, yet illusory, attempts to control them that only leads to hostile and destructive emotions and false consent. In workplaces immersed in “control-based” emotional symbolization, some people are emotionally confused about whether others are friends or foes. This situation involves constantly assessing their innocuity by verifying statements, requesting counterproofs, and monitoring behaviors, regardless of whether they are the boss, a colleague, or a client. In turn, others may demonstrate their emotions with a seemingly accepting demeanor, which may fuel mechanisms of false consent and latent conflicts within the workplace, meaning that people are ready to explode.

*Provoking* is a feature of asymmetrical relationships among parties that is rooted in emotional expectations of unlimited resources and boundaryless power, as well as the complementary refusal of limits, which only fosters irritation towards impositions and perpetuates tension and conflict. In a workplace immersed with “provoke” emotional patterns, some people (bosses, colleagues, clients) are not able to stay within the limits of the conventional rules and instead substitute them with arbitrary and self-referential rules to elicit reactions (e.g., by reminding the provocateur of the conventional rules they are violating). However, this only serves to satisfy their control over others. Others, in turn, may either patiently tolerate their behavior or react aggressively. In either scenario, the organization tends to function based on ongoing submission tests or conflicts, draining energy from productive activities.

*Obliging* is a contextual emotion featured in asymmetrical relationships among parties that is rooted in emotional deprivation and self-censoring of intolerable desires to possess others, wherein attempts to make others conform to similar sacrifices and deprivations only unfold a relational context based on paralyzing rules and constraints. In a workplace immersed in obliging emotional patterns, individuals may continuously make sacrifices, exemplarily fulfilling their work obligations, demanding others to do the same out of solidarity, and inducing guilt in those who do not comply. Others, in turn, may comply, initiating a cycle of mutual obligations that floods the working context with constraints, or defensive barriers against tasks beyond their own competence (e.g., it is not really up to me).

*Distrust* is characterized by asymmetrical relationships among parties that are rooted in the fear of others’ potential dangerousness, leading to distrusting people treating everyone as an enemy, with the instigation of doubt, uncertainty, and suspicion that systematically discards any reassurances, generates irritation, and paralyzes social dynamics. Within a “distrusting” workplace, some people struggle to discern friends and foes, and treat everyone (boss, colleagues, users) as enemies or people who should be aprioristically distrusted. Others, in turn, may uselessly attempt to reassure the distrustful people by proving them with reassurances and credentials, but their efforts only heighten suspicion, leading to stagnant working environments, paralyzed by a fear of novelty and diversity in terms of the reality.

*Complaining* involves a three-party setting and is featured in asymmetrical relationships among parties that are rooted in anger over having lost control over a third (absent) party. It involves one party (actively) seeking a second party to vent their anger, abandonment, and delusion, and instrumentally forming an alliance that requires passive, uncritical acceptance. In turn, this generates an interpersonal context of immobilized venting, whereby everything is criticized, but nothing is changed, maintaining a stagnant status quo. In “complaint-based” workplaces, some people (bosses, colleagues, users) struggle with relinquishing control over third parties or situations; they often vent their angry or disappointment by involving others in their complaints about what has gone wrong. However, the listener should remain passive, refraining from proposing solutions, as the complaint stems from their reluctance to directly confront the critical situation. These dynamics tend to shape the organizational functioning of the business, whereby everything seems to be in motion and is criticized, yet everything remains unchanged.

*Worrying* involves a similar three-party dynamic (albeit passively shaped), marked by asymmetrical relationships among parties, which is rooted in the emotional experience of worry and impotence delusion of having lost control over a third (absent) party, and persistently harboring doubts and suspicions. It involves a person venting their disappointment with a second party, who is expected to share doubts and suspicions that prevent individuals from exploring new elements and causes novelty aversion and immobilism. In a workplace immersed in worry, some people cannot tolerate the sorrow of losing control over third parties or situations. They may involve others by instilling doubts and suspicions in them towards those who display new or unusual behaviors. Others, in turn, can be infected by the unspecific sense of concern expressed, leading them to share a fear of the unknown. This fosters a defensive unity against novelties in the workplace, resulting in a paralyzing lack of initiative.

In sum, the seven negative ways to emotionally relate to organizational contexts have in common an emotional distancing from anything that is unknown, but display different forms of dysfunctional relational dynamics. Thus, toxic contextual emotions prevent individuals from fully engaging in relating positively with others and being an effective part of social processes within organizations.

On the other hand, *exchanging* is the only functional, contextual emotion that is characterized by balanced and symmetrical relationships. It builds on emotional openness to the unknown and a peaceful curiosity towards the unfamiliar, thus promoting active exploration and openness towards anything that is new, and creating a relational context that encourages interpersonal exchange and mutual adaptation. In workplaces immersed in exchange patterns, people do not make presumptions about others, even when they find them familiar, due to shared similarities. When one party views the other as an unfamiliar entity, it prompts active interactions between the parties to uncover potential contributions to the relationship. Doing so establishes the foundation for exchanging information and exploring emotional connections that bind relationships. This creates a relational dynamic at work, based on evolved dialogues and shared definitions of common rules and co-existence styles.

Overall, assessing both positive and negative contextual emotions may provide a comprehensive picture of the relational, shared dynamics in the workplace and offer a lens to understanding what creates a unified and emotionally meaningful representation of employees’ subjective experiences of working life and the social organizational environment. According to the literature, the difference between functional (i.e., adaptive, close to reality, self-enhancing, fostering cognition) and dysfunctional (i.e., maladaptive, less in touch with reality, self-defeating, impairing cognition) emotions depends on both their quantity (i.e., intensity) and their quality (David et al., 2005). For example, negative emotions could be both dysfunctional (e.g., anxiety, depression, anger, guilt, hurt), but also functional (e.g., concern, sadness, annoyance), depending on the type and intensity of the affective experience, just as positive emotions could be functional (e.g., joy, happiness, satisfaction), but also dysfunctional, if experienced at excessive levels (e.g., euphoria).

Latent profile analysis (LPA; Morin & Marsh, 2015) can be used to identify consistent patterns of variables that compose quantitatively and qualitatively distinct configurations that are experienced by homogeneous subpopulations of people. As such, LPA applied to contextual emotions qualifies as a key methodology to unfold integrated patterns of dysfunctional, or functional, emotional dynamics, emerging from the interplay of multiple simultaneous contextual emotions (i.e., quality), experienced at different levels (i.e., quantity). To date, no study has assessed whether subpopulations of employees may experience different levels of both positive and toxic contextual emotions that shape different configurations of emotional patterns of workplace behavior. Hence, the main aim of the current study is to identify distinct profiles of employees, based on the levels of different types of shared contextual emotions they experience. Given the lack of previous findings as a result of LPA applied to contextual emotions, we adopt an explorative approach to establish the profiles rooted in different configurations of emotional patterns of workplace behavior and pose specific research questions to guide our investigation, rather than developing hypotheses on the expected results. In order to assess whether distinct profiles exist, we pose the following research question:

*Research Question 1*: Are there distinct profiles of contextual emotions at work?

### Outcomes of Profiles of Contextual Emotions at Work

According to the World Health Organization (2016), 15% of working-age adults were estimated to have a mental disorder in 2019, and poor working environments pose a risk to mental health. We explored five outcomes in terms of profiles of contextual emotions at work, including two work-related variables (i.e., job satisfaction, burnout) and three health indicators (i.e., sleep disturbances, mental health, and physical health). Indeed, employee well-being within organizations depends on the quality of the social relationships and emotions experienced therein. The literature suggests that individuals are endowed with brain networks for social thinking and interpersonal relationships have a significant impact on mental and physical health, as positive social interactions help build biological systems that may protect against the adverse effects of stress (Gable & Gosnell, 2011; Umberson & Montez, 2010). Moreover, Seligman (2011) noted that while social relationships do not guarantee happiness, happiness does not often occur without positive social connections (Diener & Seligman, 2002) that provide energy to individuals and to the organization in which they work, as opposed to negative relationships that may deplete energy and lead to employee and organizational floundering (Dutton & Ragins, 2007). A previous field study on contextual emotions (Carli & Paniccia, 2002) found that professionals in a social services setting who were worn out by a “distrusting” contextual emotion pattern, during a training intervention, gained an awareness of the specific source of the fear of novelty that fueled their experience of threat in the particular context, which emancipated them from these stereotypes and enabled them to develop a new exchange-oriented emotional symbolization (i.e., positive contextual emotion) of their relationships with their patients, colleagues, and supervisors, thus improving the quality of their socio-emotional experiences at work and their related well-being.

Job satisfaction is defined as a pleasant feeling associated with work-related aspects (e.g., supervision, colleagues, promotion) that provides individuals with gratification (Locke, 1976; Spector, 1985). Such a sense of satisfaction of a fulfilled need represents an attitude towards work that contains evaluative and affective components (Landy & Conte, 2004). Indeed, the literature suggests that employees’ quality of life and job satisfaction are associated with a positive personal equilibrium within organizational interactions and the presence of positive emotional states and satisfying relationships within the work environment (Isen, 1987; Warr, 1999). In a study on contextual emotions in a public healthcare setting, Carli and Paniccia (2011) identified different clusters of context-specific organizational features that employees (doctors) associated with specific and different types of relational and emotional patterns of behavior (i.e., contextual emotions). The findings suggested that higher levels of exchanging emotional patterns of workplace behavior in regard to the employee’s relationship with their own patients and supervisors were associated with higher job satisfaction of the healthcare professionals. An additional study in a nationally representative healthcare setting (Carli et al., 2008) found that employees who perceived more negative and dysfunctional contextual emotions (e.g., claiming, distrusting, obliging) were highly dissatisfied with their job, whereas positive contextual emotions structured around “exchange” emotional patterns of workplace behavior were associated with a higher level of employee positivity and job satisfaction.

While, according to our knowledge, no study has previously examined the association of all the seven negative contextual emotions (e.g., claiming, distrusting, controlling) and the positive contextual emotion (i.e., exchanging) to employee well-being or stress-related outcomes, based on the above arguments and consistent with the broaden-and-build theory (Fredrickson, 2004), we may expect that groups of employees experiencing functional contextual dynamics would report the highest levels of job satisfaction and mental and physical health, and the lowest levels of burnout and sleep disturbances.

Hence, we pose the following research question:

*Research Question 2*: Will the functional profiles of contextual emotions relate to higher levels of (a) mental health and (b) job satisfaction, and lower levels of (c) job burnout (i.e., exhaustion, cynicism), (d) sleep disturbances, and (e) health complaints?

## 3. Method

### 3.1. Participants and Procedure

The initial sample consisted of 801 Italian workers. In regard to the analyses, we retained the participants who answered at least 4 quality check questions correctly, leading to a final sample of 659 employees. Most participants were female (59.60%), worked full-time (82.7%), and had a permanent employment contract (73.7%). The educational level of the respondents was mainly college (54.6%) and high school (38%) levels, while 3.7% of the respondents were at the junior high level. On average, the respondents were 43.56 years old (*SD* = 12.38) and had a job tenure of 11 years (*SD* = 9.50). Sixty percent of the respondents worked for organizations in the public sector and the majority (55.6%) carried out their work in-person and on-sight. The related organizations belonged to the following industry sectors: education (19.6%); healthcare (16.7%); communication and technology (13.4%); commerce (9.9%); services and finance (8.1%); transportation (3.7%); military (2.6%); manufacturing (2.0%); construction (1.7%); artistic (0.5%); agriculture (0.2%), and approximately twenty-one percent of respondents did not specify the sector in which they worked.

The survey data were anonymous and collected cross-sectionally, via Survey Monkey, from a sample of Italian adult workers. The sampling technique was based on a convenience sample strategy. The research team contacted potential participants belonging to the same organization to request their participation in the study, based on the opportunity and accessibility of the study’s participants. Participation was voluntary, anonymous, and not rewarded by an incentive. The study followed the guidelines on research ethics in compliance with the ethical principles in the Helsinki Declaration of 1964, in order to protect individual participants from any form of potential physical and/or emotional harm. The research team approached individual employees to request their participation in the study, provided them with information about the project, encouraged participation, and addressed the concerns from potential participants. The participants were provided with informed consent forms that explained the anonymous nature of the data collection and their rights as research participants.

### 3.2. Transparency and Openness

The rationale for data exclusion is reported above. The covariance matrix and analysis code are available upon request to the first author. The row data for this study are not available, as we do not have permission from the participants for row data sharing.

### 3.3. Measures

**Contextual Emotions at Work**. The Contextual Emotions at Work Scale (CEWS) is a self-reporting instrument developed by the authors. The development of the CEWS is theoretically grounded in the workplace contextual emotion aspects derived from the above review of the literature and developed in order to assess emotion-related schemes of thinking and acting that are shared among employees, each entailing specific emotions that feed working relationships. Thirty-seven items were developed to measure seven *negative* contextual emotions (i.e., claiming, controlling, provoking, distrusting, obliging, complaining, and worrying) and one *positive* contextual emotion (i.e., exchanging). Using a referent-shift approach that focuses the referent in relation to each item to contextual factors (Wallace et al., 2016), the respondents were asked to rate the frequency with which the proposed behavioral situations, associated with different emotional dynamics, occurred in their organizational context. All the items were answered based on a 5-point Likert scale, ranging from *never* (1) to *always* (5).

The common lead-in to all the scale items was: “*Within my work environment, individuals*: …”. Examples of items listed in regard to the eight sub-dimensions are: claiming (5 items) “… *expect things because they believe they are superior to others*”; controlling (5 items) “… *put a lot of effort into controlling things that happen around them*”; provoking (5 items) “… *behave according to their own rules rather than to conventional rules*”; distrusting (4 items) “… *treat each other with mistrust and never let down their guard*”; obliging (4 items) “… *do things only to fulfil obligations*”; complaining (4 items) “… *continue to complain regardless of the solutions proposed*”; worrying (5 items) “… *often talk about the impossibility of confronting things/situations*”; and exchanging (5 items) “… *are open to all types of reciprocal exchange (information, experiences, etc.)*”. Items were coded such that higher scores reflect greater levels of the specific contextual emotion they measure. The CEWS is available upon request to the first author for research purposes.

**Job satisfaction**. Job satisfaction was measured using the three items proposed by the Michigan Organizational Assessment Questionnaire’s Job Satisfaction Subscale (Cammann et al., 1983). A sample item was “*Overall, I am satisfied with my job*”. Items were answered based on a 7-point Likert scale, ranging from *strongly disagree* (1) to *strongly agree* (7).

**Job Burnout**. The Italian version (Borgogni et al., 2005) of the Maslach Burnout Inventory General Survey (Schaufeli et al., 1996) was used, including five items measuring exhaustion (e.g., “*I feel emotionally drained from my work*”) and five items assessing cynicism (“*I have become more detached from my work*”). The items were rated based on a 7-point frequency scale, ranging from *never* (0) to *daily* (6).

**Sleep disturbances**. Sleep disturbances were measured using six items from the Italian version of the Karolinska Sleep Questionnaire (Kecklund & Akerstedt, 1992), previously validated in Italy (Petitta et al., 2023). A sample item is: “*difficulties falling asleep*”. The participants were asked to indicate the frequency that they experienced sleep disorders, using a 5-point Likert scale, ranging from *never* (1) to *very often* (5).

**Health complaints**. The physical health of the respondents was measured using Hanisch’s (1992) Health Conditions Index, a formative scale that tallies the total number from 13 health conditions (e.g., “severe headaches”) experienced by respondents. The scores were summed up for each participant. The response format was coded as 0 = No and 1 = Yes, such that higher numbers indicate more psychosomatic symptoms and worse physical health.

**Mental health**. Fifteen items from Veit and Ware’s scale (Veit & Ware, 1983) were used to assess the respondents’ psychological distress. A sample item was “*During the past month…—How often have you been a very nervous person?*”. All the items were responded to using a 5-point Likert-type frequency scale, ranging from *none of the time* (1) to *all of the time* (5). The items were reverse coded, such that higher scores reflect greater levels of mental health.

### 3.4. Analytical Strategy

All the analyses were conducted using Mplus version 8 (Muthén & Muthén, 1998/2015), with robust maximum likelihood estimators (MLR). As a premise, using different steps for the diverse scales used in our study did not affect the interpretation of our findings, given that the standardized variables used in the analyses enabled the direct comparability of the results (Cohen et al., 2003; Tabachnick & Fidell, 2019). Exploratory Factor Analysis of the eight latent variables in the CEWS (i.e., seven dysfunctional and one functional contextual emotion) was performed to assess whether the items had an impact on the hypothesized latent factor. As a preliminary step, we tested a measurement model to evaluate the distinctiveness of the latent constructs in the present study and the dimensionality of the CEWS. Specifically, we performed a set of confirmatory factor analyses (CFAs), consisting of the hypothesized eight latent variables in the CEWS (i.e., seven dysfunctional and one functional contextual emotion) and the five latent outcomes (i.e., job satisfaction, emotional exhaustion, cynicism, sleep disturbances, mental health), whereas the health complaint scale, a formative measure, was excluded. Overall, we compared several alternative factorial structures, including: (a) the hypothesized 13-factor model (Model 1), (b) multiple alternative 12- and 11-factor models that combined and merged different dysfunctional contextual emotions (Model 2–8), (c) a 7-factor model (Model 9) merging all the dysfunctional contextual emotions together, (d) a 6-factor model (Model 10) merging all the CEWS items together, and (e) a 1-factor model (Model 11) merging all the 13 latent variables together (Harman, 1967). We selected the best fitting factor model and used it for the following LPA analyses.

Second, a three-step LPA approach (Asparouhov & Muthén, 2014) was implemented to extract the profiles of contextual emotions at work. To select the best fitting profile solution, we considered both multiple fit values and content decision criteria. Specifically, we used a stepwise approach to determine the number of latent profiles, starting with an LPA with two profiles and successively adding profiles (from *k* = 2 up to *k* = 6; Nylund et al., 2007). During each step, we examined seven fit information criteria, including the log likelihood (LL), sample size-adjusted bootstrap likelihood ratio test (SABIC), bootstrap likelihood ratio test (BLRT), Lo–Mendell–Rubin likelihood ratio test (LMR), Akaike information criterion (AIC), Bayesian information criterion (BIC), and entropy value. While there are no established benchmarks for LPA fit statistics, it is generally recommended that the LL, AIC, BIC, and SABIC should be lower when compared to other models. In contrast, the entropy value should be higher. Moreover, the LMP and BLRT should be statistically significant, with a *p* < 0.05. Additionally, the elbow plots of the AIC, BIC, and SABIC values were used as further information for determining the optimal number of profiles in the study sample, wherein the inflection point in the plots suggests that the optimal solution has been reached (Morin et al., 2011). Finally, using the posterior distribution from the prior step (Asparouhov & Muthén, 2014), we identified the most probable class membership, establishing the profile that an individual is most apt to fit into. Based on the guidelines by Spurk et al. (2020), we applied MLR estimation and the full information maximum likelihood estimation (FIML) approach. To prevent local maxima solutions, we set the number of random starts to 7000 and the final stage optimizations to 200 (Hipp & Bauer, 2006; for the order of the decision steps see Ram & Grimm, 2009).

Third, we employed the Mplus DE3STEP command to test the outcomes of the contextual emotion profiles (Asparouhov & Muthén, 2014).

## 4. Results

### 4.1. Descriptive Statistics and Correlations

Table 1 presents the descriptive statistics, scale reliability, and intercorrelations among the study variables. The internal consistency reliability (Cronbach’s α coefficients) ranged from 0.81 to 0.92.

### 4.2. The Measurement Model

The results from the 8-factor EFA showed excellent fit indices: χ^2^ (398, N = 788) = 775.685, *p* < 0.001, RMSEA = 0.035 (0.031; 0.038), CFI = 0.98, TLI = 0.96, and SRMR = 0.015. Each item’s impact on the intended factor was substantial and significant, thus supporting the appropriateness of the eight hypothesized latent factors. As noted, we ran several CFAs to examine the 13-factor structure. However, given the number of parameters we needed to estimate, our sample size (*N* = 659) was relatively small, violating Bentler and Chou’s (1987) recommended five-to-one ratio in terms of the required sample size to the number of free parameters. To tackle this issue and reduce the number of estimated parameters, we used item parceling for the mental health scale, because it has a significantly high number of items (15 items). Specifically, each parcel was created by averaging three items, which were sequentially assigned based on the highest to lowest item-total corrected correlations (Little et al., 2013). Apart from improving fit factor structures in regard to smaller samples, research shows that parceling may also diminish the chance of Type II errors and sampling error (Little et al., 2022). Table 2 shows the CFA results for the multiple alternative factor structures tested. As can be seen, the 13-factor model was the best-fit model, supporting the distinctiveness of the CEWS dimensions and its distinctiveness from other variables, and was thus retained for the subsequent latent profile analyses.

### 4.3. Latent Profile Analysis and Well-Being Outcomes

We used LPA to test whether there were quantitatively and qualitatively distinct contextual emotion profiles. Table 3 shows the fit statistics for the possible latent profile structures. As noted, the selection of the “best” solution depends on several features: a low AIC, SABIC, and BIC relative to other profiles, a relatively high entropy value (>0.80), a significant LMR and BLRT, and theoretically logical profiles.

**Figure 2:**
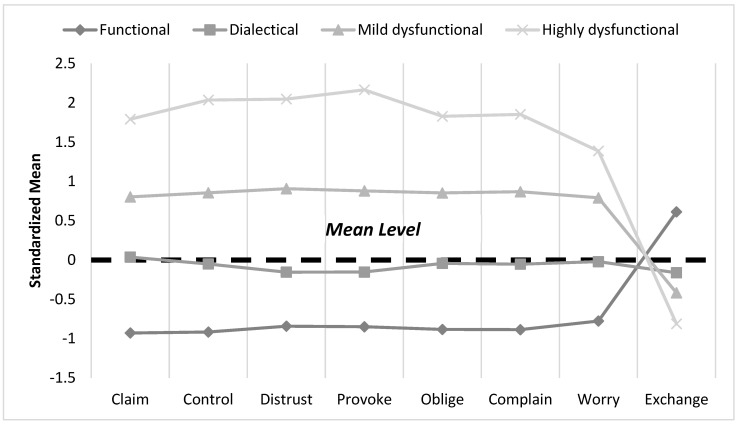
Standardized mean for the indicators of the contextual emotions (seven negative and one positive) for the *k* = 4 latent profiles. High scores for toxic contextual emotions (e.g., claiming) combined with a low score for the positive contextual emotion (exchange) indicate dysfunctional profiles, whereas low scores for toxic contextual emotions (e.g., claiming) combined with a high score for the positive contextual emotion (exchange) indicate functional profiles.

We first considered the two-profile solution, which showed the highest entropy value and a nonsignificant adjusted LMR test when including three profiles. However, the SABIC and AIC values kept descending with the inclusion of additional profiles. We decided to choose the four-profile solution because it exhibited lower LL, AIC, BIC, and SABIC values, as well as significant LMR and BLRT values, in comparison to the two- and three-profile solutions. Although the five- and six-profile solutions had slightly lower LL, AIC, BIC, and SABIC values in comparison to the four-profile solution, their entropy values were lower. Finally, the four-profile solution included four different profiles of theoretical interest that were relatively different in regard to their content (see Figure 2) and this solution was also supported by the scree test of the information criteria (see Figure S1 of the Supplementary Materials).

Table 4 shows the mean of the contextual emotion indicators for each profile and the counts in terms of each profile for the sample. We labeled those with the most positive profile as “functional contextual emotion dynamics” (*n* = 218, 33%), showing low levels of toxic contextual emotions (*M_claim_* = 1.63; *M_control_* = 1.46; *M_distrust_* = 1.35; *M_provoke_* = 1.30; *M_oblige_* = 1.40; *M_complain_* = 1.47; *M_worry_* = 1.57) and high levels of positive exchanging dynamics (*M_exchange_* = 3.54). The second group, termed “dialectical contextual emotion dynamics” (*n* = 250, 38%), was characterized by medium levels of toxic contextual emotions (*M_claim_* = 2.65; *M_control_* = 2.31; *M_distrust_*= 1.98; *M_provoke_* = 1.91; *M_oblige_* = 2.16; *M_complain_* = 2.34; *M_worry_* = 2.19), as well as medium levels of exchanging dynamics (*M_exchange_* = 2.82). We called the third group the “mild dysfunctional contextual emotion dynamics” profile (*n* = 146, 22%), because it showed moderately high levels of toxic emotions (*M_claim_* = 3.46; *M_control_* = 3.21; *M_distrust_* = 2.96; *M_provoke_* = 2.82; *M_oblige_* = 2.96; *M_complain_* = 3.31; *M_worry_* = 2.85) and moderately low levels of exchanging dynamics (*M_exchange_* = 2.58). The fourth group was called “highly dysfunctional contextual emotion dynamics” (*n* = 45, 7%), because they reported extremely high toxic emotions (*M_claim_* = 4.51; *M_control_* = 4.37; *M_distrust_* = 4.01; *M_provoke_* = 3.95; *M_oblige_* = 3.84; *M_complain_* = 4.34; *M_worry_* = 3.34) and extremely low levels of exchanging dynamics (*M_exchange_* = 2.21). Overall, we identified four latent profiles: “functional dynamics” (low toxic emotions and high exchange), “dialectical dynamics” (co-existence of medium toxic emotions and medium exchange), “mild dysfunctional dynamics” (moderately high toxic emotions and low exchange), and “highly dysfunctional dynamics” (extremely high toxic emotions and extremely low exchange). As such, in response to Research Question 1, we identified quantitatively and qualitatively different contextual emotion profiles in the sample.

The average latent profile probability for the most likely profile was 0.97, 0.92, 0.96, and 0.97, respectively, for “functional dynamics”, “dialectical dynamics”, “mild dysfunctional dynamics”, and “highly dysfunctional dynamics” profiles, indicating a very high degree of separation between these four profiles. Moreover, the employees classified as the “highly dysfunctional dynamics” profile (*M* = 48.05; *SD* = 10.88) were significantly older than those classified in regard to the “functional dynamics” profile (*M* = 42.48; *SD* = 12.76), those classified according to the “dialectical dynamics” profile (*M* = 43.98; *SD* = 12.71), and those classified according to the “mild dysfunctional dynamics” profile (*M* = 43.10; *SD* = 11.97), *F* (3, 640) = 2.65, *p* = 0.048. Additionally, employees working in-person, compared to those working remotely, were more likely to be classified according to the “mild dysfunctional dynamics” and “highly dysfunctional dynamics” profiles (χ^2^_(3)_ = 9.249, *p* < 0.05), whereas employees working for private organizations, compared to those working in the public sector, were more likely to be classified according to the “functional dynamics” profile (χ^2^_(3)_ = 13.043, *p* < 0.01).

Before conducting tests of our hypotheses, we examined whether statistically significant differences existed between workers holding different degrees of responsibility (i.e., supervisors vs. employees) with regard to contextual emotions and the related health outcomes. To ascertain this, we conducted a multivariate analysis of variance (MANOVA), with the hierarchical level (i.e., supervisors vs. employees) assigned as the within-subjects factor. When contrasting supervisors’ and employees’ scores for the eight contextual emotions and six outcomes (i.e., exhaustion, cynicism, sleep disturbances, health complaints, mental health, satisfaction), ten out of the twelve factors were not significant, thus suggesting that emotional environments do not depend upon different degrees of responsibility.

Table 5 shows the outcome levels for the four contextual emotion profiles. The “functional dynamics” profile reported the best outcomes, including significantly higher job satisfaction and mental health, as well as significantly lower emotional exhaustion, cynicism, sleep disturbances, and health complaints, in comparison to the other three profiles. The “dialectical dynamics” profile had the second-best outcomes, including significantly higher job satisfaction and mental health, as well as significantly lower emotional exhaustion, cynicism, sleep disturbances, and health complaints than those reported in regard to the “mild dysfunctional dynamics” and “highly dysfunctional dynamics” profiles. On the other hand, the “mild dysfunctional dynamics” profile reported significantly higher job satisfaction and mental health, as well as significantly lower emotional exhaustion, cynicism, sleep disturbances, and health complaints than the “highly dysfunctional dynamics” profile. However, there was no significant difference in regard to mental health in terms of the “mild dysfunctional dynamics” and “highly dysfunctional dynamics” profiles. Overall, employees in the dialectical, mild dysfunctional, and highly dysfunctional groups reported progressively higher levels of poor health outcomes and progressively lower levels of satisfaction, whereas the functional group was at low risk of stress and reported to be the most satisfied in their job. These results lend support to the relevance of Research Question 2 (a, b, c, d, e) and indicate that different contextual emotion profiles at work relate to different levels of job satisfaction, burnout, sleep disturbances, health complaints, and mental health.

## 5. Discussion

Poor interpersonal relations combined with toxic emotions and a lack of practices related to respect for workers are psychosocial hazards within an organization that significantly contribute to workplace distress (Stoewen, 2016). Within the framework of intertwined emotional and relational factors that orient mental representations of the work experience shared among employees, the current paper proposed the novel conceptualization of contextual emotions at work that complements existing emotion-related models by combining relational and emotional processes. The aim was to identify distinct profiles among employees or subpopulations who may differ in terms of the level of positive (e.g., exchanging) and toxic (e.g., controlling, claiming, provoking) emotional ways of symbolizing their workplace, and assess how such emergent latent profiles may differentially relate to employee work-related and health outcomes.

The LPA revealed four distinctive latent profiles. Specifically, we found that a “functional dynamics” profile exists for employees (33%) who report low (below average) levels of all seven toxic emotions and a high (above average) level of the positive exchange symbolization, suggesting an overall *positive* configuration of emotional patterns of workplace behavior. The “dialectical dynamics” profile (38%) is characterized by average levels of all seven toxic emotions and average levels of exchange symbolization, suggesting a simultaneous and *dialectical* co-existence of medium negative dimensions, in combination with a medium positive dimension. It is worth noting that we coined the term “dialectical” (i.e., related to opposing forces), because while all the other profiles include a pattern of contextual emotions wherein positive and negative dimensions are skewed and unevenly distributed, the dialectical profile displays the same levels of opposite dimensions (e.g., toxic and positive contextual emotions) and, thus, suggests the co-occurrence of “opposite forces”. A third “mild dysfunctional dynamics” profile (22%) reported high levels of all seven toxic emotions (above average) and low levels of exchange symbolization (below average), suggesting a polarized *negative* configuration of highly negative contextual emotions, in combination with a low positive contextual emotion. A final “highly dysfunctional dynamics” profile (7%) reported very high levels of all seven toxic emotions (above average) and very low levels of exchange symbolization (below average), suggesting an extremely negative configuration of highly negative contextual emotions, in combination with a low positive contextual emotion. Thus, the last two profiles are qualitatively similar, but are quantitatively different (i.e., they have the same shape in regard to all the dimensions, but at different levels, across the two groups). Moreover, the average latent profile probability result was very high for all of the groups, thus supporting a high degree of separation between these four profiles and the final *k* = 4 LPA solution, reflecting four distinct emergent organizations of emotional patterns of workplace behavior, uniting the members of each subgroup.

Given that one of the most critical issues in regard to LPA is the identification of “true” versus “spurious” profiles (Spurk et al., 2020), we reflect on our decision regarding the four profiles. A three-profile solution, including mild and highly dysfunctional profiles and the functional profile, but not the dialectical (average) profile, may also be viable. However, we believe that a dialectical profile is of theoretical and practical relevance in order to capture a nuanced picture of workplace emotional dynamics. On the one hand, the results show that when toxic contextual emotions (e.g., claiming, provoking, controlling) are experienced at medium levels, they are likely to co-exist and dynamically swing according to positive symbolizations (e.g., “exchanging”). On the other hand, capturing a dialectical (average) profile unravels the unhealthy effects of “average” toxic dynamics, the harmful effects of which might go unnoticed and be underestimated, while also helping to avoid polarized views of organizational mechanisms. Moreover, our findings on the significant differences among the mean levels of the health outcomes further support the relevance and discriminant usefulness of the four-profile solution.

Notably, the probability of profile membership was similar across the three main blocks of positive (i.e., 33% of workers experiencing “functional dynamics”), average (i.e., 38% experiencing “dialectical dynamics”), and negative (i.e., 22% experiencing “mild dysfunctional dynamics” and 7% experiencing “highly dysfunctional dynamics”) profile types, thus showing a distribution of roughly one third of employees for each block (the “dialectical dynamics” profile being the largest group) and an overall balanced distribution of positive, average, and negative dynamics among workers. Moreover, the results on the demographics and job characteristics of the sample suggest that the employees classified according to the “highly dysfunctional dynamics” profile were the oldest and those classified in the “mild” and “highly dysfunctional dynamics” groups had in-person working arrangements, whereas employees classified according to the “functional dynamics” profile worked for private organizations. Our findings comport with the literature (e.g., Schein & Maanen, 2016), suggesting that longer-tenured employees are more deeply embedded within the organizational functioning of the business and, therefore, tend to reinforce even its more negative relational mechanisms (e.g., toxic contextual emotions). Similarly, working on-site can facilitate more frequent interactions and foster stronger connections that may reiterate problematic socialization processes and their crystallization over time (Bilderback & Kilpatrick, 2024).

Examining the associations of latent profiles with employee outcomes also highlights how outcomes differ as a function of profile membership. Specifically, we found that those employees classified according to “dialectical”, “mild dysfunctional”, and “highly dysfunctional” dynamic profiles reported increasingly higher levels of burnout, sleep disturbances, poor physical and mental health, and increasingly lower levels of job satisfaction, whereas the “functional dynamics” group was the most satisfied with their work and at low risk of health problems. That is, employees who share emotional patterns of behavior rooted in a combination of high negative (e.g., controlling) emotional dynamics that trump positive (e.g., exchanging) emotional dynamics (as is the case of those classified according to dysfunctional dynamics profiles) tend to experience toxic relational environments that foster their sense of discomfort and eventually lead to physical and psychological distress, as well as dissatisfaction in work situations. These findings align with research on the combination of positive and negative affectivity, which focuses on intra-individual emotional processes and adopts a variable-centered approach, suggesting that a positive discrepancy (i.e., a preponderance for positive affectivity over negative affectivity) is a significant predictor of high employee well-being (Yoon et al., 2021). While our findings generally comport with the affect-related literature demonstrating how positive emotion-related collective phenomena (e.g., positive team affective climate, Gamero & González-Romá, 2020; emotional culture of compassion, Barsade & O’Neill, 2014) are associated with higher employee well-being (e.g., satisfaction, low burnout), the overarching framework of our study goes further and adds information on which specific subgroups of employees are associated with higher well-being as opposed to more at-risk subpopulations. Overall, our findings contribute to complement existing emotion-related models by combining relational and emotional processes, and provides an overarching framework that allows for the identification of differently vulnerable groups of employees in terms of the unwanted consequences of toxic emotional environments. In so doing, our approach departs from traditional models in that it: (a) combines relational and emotional processes in order to explain emotion-related workplace behavior, (b) shifts the focus from an intra-individual approach towards an inter-individual perspective in studying affect phenomena in organizations, and (c) provides a methodological approach (i.e., LPA) that enables the examination of whether an affect-related phenomenon is heterogeneously experienced within a population of employees and identifies specific subgroups, each displaying a unique pattern of the affect experience.

### 5.1. Theoretical Implications

Our findings add to the literature on affect in organizations in several ways. First, we contribute to theories on emotions as collective phenomena (e.g., Barsade & O’Neill, 2014; Elfenbein, 2023; Gamero & González-Romá, 2020; Gross, 2015), by introducing the concept of contextual emotions at work as emotional and relational processes that orient the mental representations of work experiences shared among employees (i.e., emotional symbolizations of reality; Carli & Paniccia, 2003). The research on workplace dynamics tends to suggest that the effects of emotions among employees may differ depending on whether one takes an intrapersonal or interpersonal perspective (e.g., Ashkanasy, 2003; Cheshin, 2020). Moreover, to date, much of the research on emotion dynamics tends to focus on evaluating specific features of emotions separately, despite the recognition that it may be simplistic to assume that people respond with single discrete emotions during interpersonal exchanges. To complement existing theoretical perspectives (e.g., Ashkanasy & Dorris, 2017), we propose contextual emotions at work that shift the focus from an intra-individual towards an inter-individual perspective in studying collective affect-related phenomena in organizations, while also combining relational and emotional processes in order to explain emotion-related workplace behavior. For example, research on the affective climate of teams (Gamero & González-Romá, 2020) or emotional culture (Barsade & O’Neill, 2014) intersects with how emotions are shared among employees. Yet, they aggregate at the collective level, in terms of an employee’s individual perception of team members’ or other employees’ emotional expression (e.g., discrete emotions) or feelings. Alternatively, contextual emotions refer to collective relational dynamics that are fueled by different types of emotional experiences arising from social interactions (e.g., fear of others’ omnipotence and intolerance of limitations), which are shared among employees and are associated with different patterns of interpersonal behaviors that stand as indicators of a specific contextual emotion. For example, the affective climate of teams or emotional culture inform us on how employees capture specific emotional or mood cues and tend to share such affective states, whereas contextual emotions reveal specific relational patterns (e.g., closed to diversity, false consent, paralyzing rules and constraints) that structure daily interactions over time, based on mutually satisfying emotional dynamics that bond employees within the workplace. In doing so, we provide: (a) a holistic model of the functioning of the mind that thoroughly addresses the conjoint interplay of cognition, emotion, and context in explaining organizational behavior and related emotion-driven relational dynamics, (b) an overarching framework explaining how the emotional atmosphere at work develops as a result of organized ways of emotionally thinking about specific aspects of the context (i.e., emotional symbolizations) that are involuntarily shared among employees and maintained by repeated interactions (Carli & Paniccia, 2003), and (c) a conceptual framework that includes two foci of emotional symbolizations (i.e., negative and positive emotional symbolizations), thus providing a balanced and nuanced understanding of emotional dynamics at work.

Second, by adopting a latent variable clustering approach (Collins & Lanza, 2013; Wang & Hanges, 2011) that focuses on identifying latent subpopulations based on a certain set of variables (Spurk et al., 2020), we identify distinct emotional dynamic profiles. That is, we supplement variable-centered approaches that consider different characteristics in isolation (i.e., negative or positive emotional symbolizations) by simultaneously examining two foci of emotional symbolizations (i.e., negative and positive) and unfolding the unobserved heterogeneity among employees in terms of the emotional ambience that they experience at work, thereby providing holistic insights into potential psychosocial hazards (Urbanaviciute et al., 2021). For example, traditional variable-centered approaches inform us about how affect-related phenomena (e.g., the affective climate of teams) tend to be associated with employees’ outcomes (e.g., satisfaction; Gamero & González-Romá, 2020) in regard to the whole employee population. Conversely, using an LPA framework enables us to identify how employee outcomes (e.g., mental health) tend to be diversely associated with specific subgroups of employees within the whole employee population, based on their differential experience of affect-related phenomenon (e.g., dysfunctional dynamic vs. functional dynamic subgroups).

Third, the current study also informs the occupational health literature. Indeed, vocational behavior research increasingly relies on LPA methodology (Cheshin, 2020) and our findings reveal that different employee profiles face varied levels of risk in terms of negative health outcomes. This indicates the theoretical importance of incorporating both positive and negative symbolizations and assessing how their combined effects are crucial for unraveling toxic configurations of emotional dynamics at work. Moreover, latent profiles that emerge from qualitatively and quantitatively different combinations of contextual emotions at work demonstrate an additional type of emotional demand (Bakker et al., 2023; Le Blanc et al., 2001) or, rather, toxic contextual emotions that require sustained emotional effort from all exposed employees and are likely associated with an increased risk of negative work and health consequences.

Finally, the present contribution builds upon the TAD (Carli & Paniccia, 2003) and extends its groundwork to the latent variable clustering approach, namely LPA, which enables the identification of different subgroups of employees with different combinations of emotional symbolizations regarding their working context and related emotional interactions. Doing so helps develop our understanding of how employees falling within these profiles may differ in regard to their vulnerability to poor work-related and health outcomes.

### 5.2. Practical Implications

Our findings are of practical significance for scholars and practitioners alike. First, by adopting LPA to identify subgroups of employees who are exposed to toxic (vs. functional) social environments and, therefore, are the most at risk of developing work-related and health issues, we map the “social fitness” of organizations and unravel the heterogeneity in psychosocial environments (Urbanaviciute et al., 2021). Our results offer guidance on how to manage emotional dynamics at work. By creating a map of areas in need of change, organizations can implement programs to promote and solidify new norms that are aligned with mindful organizational practices (DeJoy, 2005). Organizational interventions aimed at preventing emotional and relational hardships and promoting fair and respectful interpersonal practices may involve developing and implementing programs inspired by principles of two-way communication, negotiation, constructive feedback, and respectful performance management to cultivate healthy social environments (Stoewen, 2016). These interventions serve as measures to combat dysfunctional contextual emotions, marked by a lack of consultation, situations involving conflict and harassment, intolerance in regard to diversity, and a lack of support, dignity, or respect (World Health Organization, 2016).

Second, the literature on emotions suggests that a positive discrepancy (i.e., a preponderance for positive affectivity over negative affectivity) not only predicts employee health (physical and mental) and satisfaction, but that the most important path to well-being is via increased positive affectivity, and, subordinately, via attenuated negative affectivity (Yoon et al., 2021). Contextual emotions at work are the responsibility of all organizational stakeholders. Thus, to create healthy emotional and relational environments within organizations, it is necessary to raise awareness about emotional dynamics among team members (e.g., Cilliers, 2000) and the existence of emotional contagion among multiple stakeholders (e.g., Petitta et al., 2020) by involving all parties in order to effectively implement change. Towards this end, the Search Conference (Williams, 1979) qualifies as an intervention tool that engages multiple groups in a collaborative full-immersion workshop. Its primary objective is to cultivate awareness of the organization’s context and operational methods by examining its past and present. This serves as a preliminary step towards collective thinking aimed at resolving specific organizational issues or charting new directions for the future (Williams, 1979). It aims to assist stakeholders within a system by: (a) fostering a mutual understanding of their current circumstances, (b) crafting strategies for proactive change based on their experiences, and (c) deliberating on the steps required to implement the agreed-upon changes. Thus, ideally all members involved in dysfunctional contextual dynamics (e.g., employees, supervisors) should actively participate in collectively identifying the areas in need of change (Williams, 1979). They should collaborate to determine new directions and approaches to improve the emotional dynamics within the workplace and foster better workplace relationships. This process involves confronting and synthesizing diverging views to create a mutually satisfying plan, which applied to implement an intervention in regard to contextual emotions may help by collectively shaping healthier emotional ambiences in the workplace.

### 5.3. Strengths, Limitations, and Future Directions

The present paper is the first to identify vulnerable subpopulations of employees diversely exposed to toxic vs. functional configurations of emotional/relational ambience (i.e., *LPA-classified* emotional patterns of workplace behavior) and related outcomes. Moreover, our large sample size goes beyond the recommended threshold to determine the appropriate number of latent profiles (Spurk et al., 2020).

We also note several limitations. First, we employed a cross-sectional research design, with self-reporting features. Thus, the results could be somewhat skewed due to common method variance contamination (Podsakoff et al., 2003). While we are unable to determine the cause of the links between the variables of interest in our investigation, as in other correlational studies, the relationships between the contextual emotion profiles and the outcomes of interest are theoretically driven. Nonetheless, future studies may replicate our finding using 2-wave data to introduce temporal separation between our purported predictors (i.e., contextual emotion profiles) and the dependent variables (i.e., health outcomes).

Second, our research relies on information from a single source and may benefit from future designs that include multi-source data. Subsequent research endeavors may replicate the current findings with data from various sources provided by diverse informants, such as objective measures of health indicators (e.g., archive data on sick leave, absenteeism) and from supervisors (e.g., hetero-assessment of employee burnout, satisfaction, etc.). While employees themselves are arguably the best informant on their emotional experience within their social context, multi-source data based on third-party perceptions and/or archive records may strengthen our inferences about emotionally anchored organizational behavior and address issues associated with relying solely on self-reports (Batista-Foguet et al., 2019).

Third, the use of a convenience sample may cause a problem in terms of the generalizability of our findings. While our dataset includes respondents from numerous industries and employment contexts, our sample may not be representative of the actual national labor force. Thus, it is important to use caution when extrapolating our findings to the whole Italian worker population and our results should be replicated in future studies using a larger sample from additional occupational settings.

Finally, while we only examined age, organization type (i.e., public vs. private), and work arrangements (i.e., in-person vs. remote) as covariates of the outcomes experienced by our participants in association with their emotional symbolizations at work, future studies may include additional contextual variables such as organizational culture norms and values (Schein, 1985) to reveal organizational boundary conditions that enable stakeholders to model their relational environment and implement changes to the social fabric of their organization.

## 6. Conclusions

Contextual emotions at work are relational patterns shared among employees and grounded in different types of emotional processes. They can take the form of toxic emotional dynamics (e.g., claiming, controlling, distrusting, provoking) or, conversely, positive emotional dynamics (i.e., exchanging). Using latent profile analysis, our research identified within a sample of 801 Italian workers, four subpopulations of employees who experienced differing levels of both positive and negative dynamics of emotions (i.e., different configurations of emotional patterns of workplace behavior). Employees in the subgroups shared the same relational and emotional experience and the four subpopulations displayed the following patterns: “functional dynamics” (low toxic emotions and high exchange), “dialectical dynamics” (co-existence of medium toxic emotions and medium exchange), “mild dysfunctional dynamics” (moderately high toxic emotions and low exchange) and “highly dysfunctional dynamics” (extremely high toxic emotions and extremely low exchange). Moreover, the four subgroups showed a differential association with the work- and health-related outcomes, such that employees in the dialectical, mild dysfunctional, and highly dysfunctional groups reported progressively higher levels of poor health outcomes and progressively lower levels of satisfaction, whereas the functional group was at low-risk of stress and the most satisfied in regard to their job. Overall, our research complements existing emotion-related models by combining relational and emotional processes, and provides an overarching framework that allows for the identification of differently vulnerable groups of employees in terms of the unwanted consequences of their exposure to toxic emotional environments.

## Figures and Tables

**Table 1:** Descriptive statistics, correlations, and reliability.

Variable	M	SD	1	2	3	4	5	6	7	8	9	10	11	12	13	
1. Claiming	2.61	1.06	0.91													
2. Controlling	2.36	0.99	0.79 **	0.90												
3. Distrusting	2.12	0.92	0.72 **	0.79 **	0.86											
4. Provoking	2.05	0.88	0.72 **	0.78 **	0.76 **	0.89										
5. Obliging	2.20	0.90	0.69 **	0.75 **	0.70 **	0.75 **	0.84									
6. Complaining	2.40	1.05	0.68 **	0.71 **	0.71 **	0.76 **	0.71 **	0.92								
7. Worrying	2.20	0.82	0.54 **	0.60 **	0.60 **	0.61 **	0.66 **	0.74 **	0.86							
8. Exchanging	2.97	0.93	−0.38 **	−0.38 **	−0.45 **	−0.42 **	−0.34 **	−0.47 **	−0.24 **	0.90						
9. Job satisfaction	5.48	1.25	−0.36 **	−0.33 **	0.39 **	−0.37 **	−0.30 **	−0.36 **	−0.22 **	0.36	0.81					
10. Emotional exhaustion	2.10	1.41	0.40 **	0.39 **	0.38 **	0.38 **	0.41 **	0.42 **	0.36 **	−0.23 **	−0.52 **	0.90				
11. Cynicism	1.51	1.27	0.38 **	0.39 **	0.41 **	0.37 **	0.40 **	0.41 **	0.36 **	−0.30 **	−0.66 **	0.64 **	0.83			
12. Sleep disturbances	2.76	0.90	0.30 **	0.28 **	0.30 **	0.32 **	0.32 **	0.35 **	0.33 **	−0.16 **	−0.34 **	0.62 **	0.44 **	0.88		
13. Health complaints	2.32	2.19	0.38 **	0.37 **	0.39 **	0.40 **	0.35 **	0.40 **	0.35 **	−0.19 **	−0.33 **	0.53 **	0.37 **	0.58 **	−	
14. Mental health	4.34	0.86	−0.27 **	−0.29 **	−0.29 **	−0.28 **	−0.29 **	−0.31 **	−0.28 **	0.18 **	0.46 **	−0.55 **	−0.53 **	−0.58 **	−0.42 **	0.92

*Notes*: The reliability values are displayed in the diagonal direction; ** *p* < 0.01.

**Table 2:** Results of confirmatory factor analyses for the testing of the measurement model.

Model	χ^2^	*Df*	CFI	TLI	RMSEA	SRMR	Δχ^2^ (Δdf)
M_1_: 13 factors	3700.11	1575	0.912	0.905	0.045	0.071	
M_2_: 12 factors (provoke and oblige merged)	3851.21	1587	0.906	0.899	0.047	0.072	151.1(12) ***
M_3_: 12 factors (complain and worry merged)	3900.81	1587	0.901	0.893	0.048	0.072	200.7(12) ***
M_4_: 12 factors (control and distrust merged)	3834.48	1587	0.907	0.900	0.046	0.072	134.37(12) ***
M_5_: 12 factors (claim and control merged)	3990.04	1587	0.901	0.893	0.048	0.072	289.93(12) ***
M_6_: 12 factors (claim & distrust merged)	4076.86	1587	0.897	0.889	0.049	0.072	376.75(12) ***
M_7_: 11 factors (control, provoke, and oblige merged)	4046.32	1598	0.899	0.892	0.048	0.072	346.21(23) ***
M_8_: 11 factors (distrust, complain, and worry merged)	4483.85	1598	0.881	0.872	0.052	0.075	783.74(23) ***
M_9_: 7 factors (negative contextual emotions as one factor)	5643.26	1632	0.834	0.826	0.061	0.076	1943.15(57) ***
M_10_: 6 factors (negative and positive contextual emotions as one factor)	7002.63	1638	0.778	0.768	0.070	0.084	3302.52(63) ***
M_11_: 1 factor (contextual emotions and outcomes as one factor)	12,661.09	1652	0.545	0.529	0.101	0.116	8960.98(77) ***

*Notes:* CFI = comparative fit index; TLI = Tucker–Lewis index; RMSEA = root mean square error of approximation; SRMR = standardized root mean square residual; *** *p* < 0.001.

**Table 3:** Fit indices of profile indicators.

No. of Profiles	LL	FPs	AIC	BIC	SABIC	BLRT (*p*)	LMR (*p*)	Entropy
2	−6196.167	25	12,554.603	12,442.335	12,475.227	0.0000	0.0000	0.921
3	−5758.889	34	11,585.777	11,738.462	11,630.511	0.2467	0.2803	0.907
4	−5516.602	43	11,119.203	11,312.305	11,175.779	0.0003	0.0003	0.905
5	−5442.651	52	10,979.302	11,222.820	11,057.719	0.0169	0.0180	0.852
6	−5378.374	61	10,878.748	11,152.683	10,959.006	0.0029	0.0031	0.863

*Notes:* LL = log likelihood; FPs = free parameters; SABIC = sample size-adjusted Bayesian information criteria; BLRT (*p*) = *p*-value for the bootstrapped likelihood ratio test; LMR (*p*) = *p*-value for the adjusted Lo–Mendell–Rubin test; AIC = Akaike information criteria; BIC = Bayesian information criteria. For the best fitting model, the LL, AIC, BIC, and SABIC should be lower when compared to other models. In contrast, the entropy value should be higher. Moreover, the LMP and BLRT should be statistically significant, with a *p* < 0.05.

**Table 4:** Profile counts and contextual emotion means.

Profile	N	% of Sample	Claiming	Controlling	Distrusting	Provoking	Obliging	Complaining	Worrying	Exchanging
Functional	218	33%	1.627	1.458	1.350	1.303	1.403	1.471	1.566	3.539
Dialectical	250	38%	2.651	2.312	1.982	1.914	2.158	2.343	2.185	2.818
Mild dysfunctional	146	22%	3.463	3.206	2.958	2.819	2.962	3.309	2.849	2.581
Highly dysfunctional	45	7%	4.511	4.370	4.005	3.948	3.837	4.340	3.337	2.213

*Note:* The table shows the mean for the contextual emotion indicators for each profile and the number of subjects included in each profile.

**Table 5:** Three-step results for outcomes on contextual emotion profiles.

Mean	Functional (a) P1	Dialectical (b) P2	Mild Dysfunctional (c) P3	Highly Dysfunctional (d) P4	Chi Square	Post Hoc Tests
Emotional exhaustion	1.317 _b,c,d_	2.124 _a,c,d_	2.628 _a,b,d_	3.979 _a, b, c_	188.907	P1 < P2 < P3 < P4
Cynicism	0.797 _b,c,d_	1.493 _a,c,d_	2.099 _a,b,d_	3.113 _a, b, c_	247.074	P1 < P2 < P3 < P4
Sleep disturbances	2.312 _b,c,d_	2.794 _a,c,d_	3.132 _a,b,d_	3.558 _a, b, c_	128.759	P1 < P2 < P3 < P4
Health complaints	1.414 _b,c,d_	2.033 _a,c,d_	3.207 _a,b,d_	5.298 _a, b, c_	133.217	P1 < P2 < P3 < P4
Mental health	4.606 _b,c,d_	4.403 _a,c,d_	4.023 _a,b_	3.648 _a, b_	58.488	P1 > P2 > P3 = P4
Job satisfaction	4.652 _b,c,d_	4.405 _a,c,d_	4.232 _a,b,d_	3.744 _a, b, c_	63.750	P1 > P2 > P3 > P4

## Data Availability

The covariances matrix and analysis code are available upon request from the first author. The data in the present study are unavailable as the participants did not provide their permission to share the raw data.

## Supplementary Materials

The following supporting information can be downloaded at: https://www.mdpi.com/article/10.3390/ejihpe15070122/s1, Figure S1: Scree-test of Information Criteria for 2 to 6 Profiles LPA Solution.
