# Supplementary material for: Contextual Emotions in Organizations: A Latent Profile Analysis of Their Co-Occurrence and Their Effects on Employee Well-Being
Source: Eur J Investig Health Psychol Educ. 2025 Jul 2;15(7):122. doi: 10.3390/ejihpe15070122 (PMC12296042; doi:10.3390/ejihpe15070122)
Supplement: Supplementary file 1 [file ejihpe-15-00122-s001.zip › ejihpe-3593026-supplementary.pdf]

Supplementary Materials

Figure S1

Scree-test of Information Criteria for 2 to 6 Profiles LPA Solution

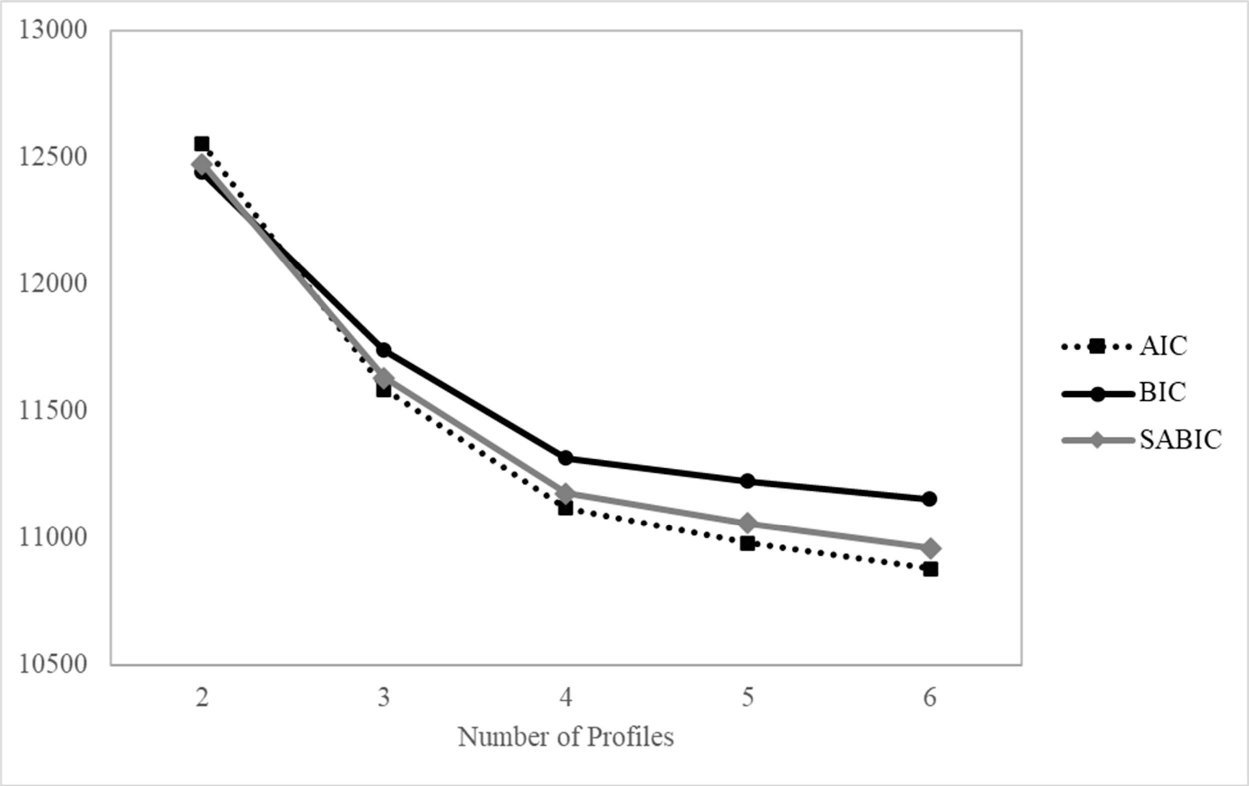

Note. AIC = Akaike Information Criterion; BIC = Bayesian Information Criterion; SABIC = Sample Size Adjusted BIC.
